# Diffuse‐Type Tenosynovial Giant Cell Tumor of the Hip With Acetabular Bone Involvement: A Case Report With Radiologic‐Pathologic Correlation

**DOI:** 10.1002/cnr2.70696

**Published:** 2026-09-18

**Authors:** Wang Quanbing, Xing Huanhuan, Zhang Xing, Yang Tao, Zhang Jun, Zhu Lei, Li Kai, Luo Lei, Ao Feng, Yang Wei

**Affiliations:** ^1^ Department of Orthopedics and Joint Surgery, Renmin Hospital Hubei University of Medicine Shiyan Hubei China; ^2^ Department of Hospital Infection Management, Renmin Hospital Hubei University of Medicine Shiyan Hubei China; ^3^ Department of Pathology, Renmin Hospital Hubei University of Medicine Shiyan Hubei China; ^4^ Department of Nuclear Medicine, Renmin Hospital Hubei University of Medicine Shiyan Hubei China; ^5^ Department of Radiology, Renmin Hospital Hubei University of Medicine Shiyan Hubei China; ^6^ Hubei Clinical Medical Research Center for Atherosclerotic Cardiovascular Diseases Shiyan Hubei China

**Keywords:** acetabular erosion, case report, diffuse‐type tenosynovial giant cell tumor, hip, histopathology

## Abstract

**Background:**

Diffuse‐type tenosynovial giant cell tumor (D‐TGCT) is an uncommon synovial neoplasm. Hip involvement is rare and may be difficult to recognize because symptoms are nonspecific and osseous erosion can mimic more aggressive infectious, inflammatory, or neoplastic processes. We report a case of hip D‐TGCT that presented as an erosive acetabular lesion and required histopathologic and immunohistochemical confirmation.

**Case:**

A 56‐year‐old man presented with acute right hip pain and restricted motion after a recent febrile illness that had partially improved following empirical intravenous cefuroxime therapy. Magnetic resonance imaging (MRI) demonstrated diffuse intra‐articular synovial proliferation with heterogeneous low‐to‐intermediate signal intensity on T1‐weighted images, heterogeneous signal intensity on T2‐weighted images, and heterogeneous enhancement after contrast administration. Computed tomography (CT) showed joint effusion and thinning and erosion of the anterior and inferomedial acetabular wall, whereas plain radiographs were unremarkable. Because the imaging and laboratory findings were inconclusive, the patient underwent surgical excision and synovectomy, curettage of the eroded acetabular wall, alcohol ablation, and autologous iliac bone grafting. Histopathologic examination demonstrated mononuclear cells, osteoclast‐like multinucleated giant cells, foamy histiocytes, chronic inflammatory cells, and abundant hemosiderin deposition. Integration of the characteristic morphology, diffuse intra‐articular growth pattern, imaging findings, and supportive immunohistochemistry established the diagnosis of D‐TGCT. Microbiologic cultures were negative. No postoperative radiotherapy was administered. At the 6‐month follow‐up, the patient reported sustained pain relief and improved hip motion, although mild restriction of motion persisted.

**Conclusion:**

Hip D‐TGCT should be considered when an intra‐articular hip lesion shows synovial proliferation with acetabular erosion, even when inflammatory findings raise concern for alternative diagnoses. Magnetic resonance imaging and computed tomography are complementary for defining soft‐tissue extent and osseous involvement, but definitive diagnosis depends on clinicopathologic correlation. This case highlights the diagnostic value of histopathology and immunohistochemistry in destructive‐appearing hip lesions.

**Abbreviations**
ALP: alkaline phosphatase
CRP: C‐reactive protein
CT: computed tomography
D‐TGCT: diffuse‐type tenosynovial giant cell tumor
ESR: erythrocyte sedimentation rate
MRI: magnetic resonance imaging
TGCT: tenosynovial giant cell tumor
WHO: World Health Organization

## 1 Background

Tenosynovial giant cell tumor (TGCT) is a synovial neoplasm arising from the synovial lining of joints, bursae, or tendon sheaths. According to the 2020 World Health Organization (WHO) Classification of Soft Tissue and Bone Tumours, TGCT is categorized on the basis of growth pattern into localized TGCT and diffuse‐type TGCT (D‐TGCT) [1]. D‐TGCT, previously termed pigmented villonodular synovitis (PVNS), is a locally aggressive lesion characterized by extensive synovial proliferation, nodular growth, and a propensity for recurrence. It most commonly affects the knee [2], finger, ankle and shoulder [3], whereas hip involvement is uncommon [4, 5].

D‐TGCT of the hip is challenging to diagnose because the joint is deeply situated and the presenting symptoms are often nonspecific. Patients may present only with pain, stiffness, or limitation of motion, and plain radiographs can be normal early in the disease course. When osseous erosion is present, the imaging appearance may overlap with infection, inflammatory synovitis, synovial chondromatosis, or other destructive acetabular lesions [4, 5, 6]. The educational value of the present case lies not in the presence of acetabular erosion alone, but in the diagnostic pitfall created by acute symptoms, equivocal inflammatory markers, history of chills and high fever, and a destructive‐appearing acetabular lesion, with definitive diagnosis established only after clinicopathologic correlation.

## 2 Case Presentation

### 2.1 Patient Information and Clinical Findings

A 56‐year‐old man presented with a 4‐day history of right hip pain and restricted motion. He was afebrile on admission, with a body temperature of 36.4°C. One week before admission, he had experienced acute chills and high fever and had received empirical intravenous cefuroxime for 3 days at a local hospital, with partial symptomatic improvement. He subsequently developed acute right hip pain that was most severe at night, disturbed his sleep, and worsened with movement. The pain did not substantially improve with rest or changes in position. There was no definite history of trauma, weight loss, fatigue, or evening low‐grade fever. Physical examination revealed marked tenderness over the right hip. Pain was provoked by internal rotation, adduction, and flexion, with mildly restricted range of motion. No local erythema, warmth, or swelling was observed, and the distal neurovascular examination was unremarkable.

### 2.2 Timeline

A CARE‐oriented summary of the clinical course is provided in Table 1.

**TABLE 1:** Clinical timeline according to the CARE case‐report structure.

Time point	Clinical event
1 week before admission	Acute chills and high fever; empirical intravenous cefuroxime treatment for 3 days at a local hospital, with partial symptomatic improvement.
4 days before admission/admission	Acute right hip pain with restricted mobility, most severe at night and worsened by movement; physical examination showed right hip tenderness and pain with internal rotation, adduction, and flexion.
Admission work‐up	MRI before and after contrast administration, CT, whole‐body bone scintigraphy, and laboratory testing were performed. MRI demonstrated right hip intra‐articular synovial tissue with heterogeneous enhancement, and CT showed erosion of the anterior and inferomedial acetabular wall.
Surgery	Surgical excision and synovectomy with curettage of the eroded acetabular wall, alcohol ablation, and autologous iliac bone grafting were performed.
Pathologic diagnosis	Histopathology and immunohistochemistry demonstrated features consistent with diffuse‐type tenosynovial giant cell tumor; joint‐fluid cultures were negative.
Postoperative day 3	Fever, mild cough, and fatigue developed; chest CT showed bilateral patchy opacities, and pulmonary infection was treated with intravenous cefuroxime and supportive care.
2‐week follow‐up	The incision was well healed; right hip pain and range of motion had improved.
2‐month follow‐up	The bone graft in the acetabular bone defect area had healed well.
6 months after surgery	The patient reported sustained pain improvement and had returned to independent daily activities and work, although mild limitation of right hip motion persisted. No postoperative radiotherapy was administered.

### 2.3 Diagnostic Assessment

**FIGURE 1:**
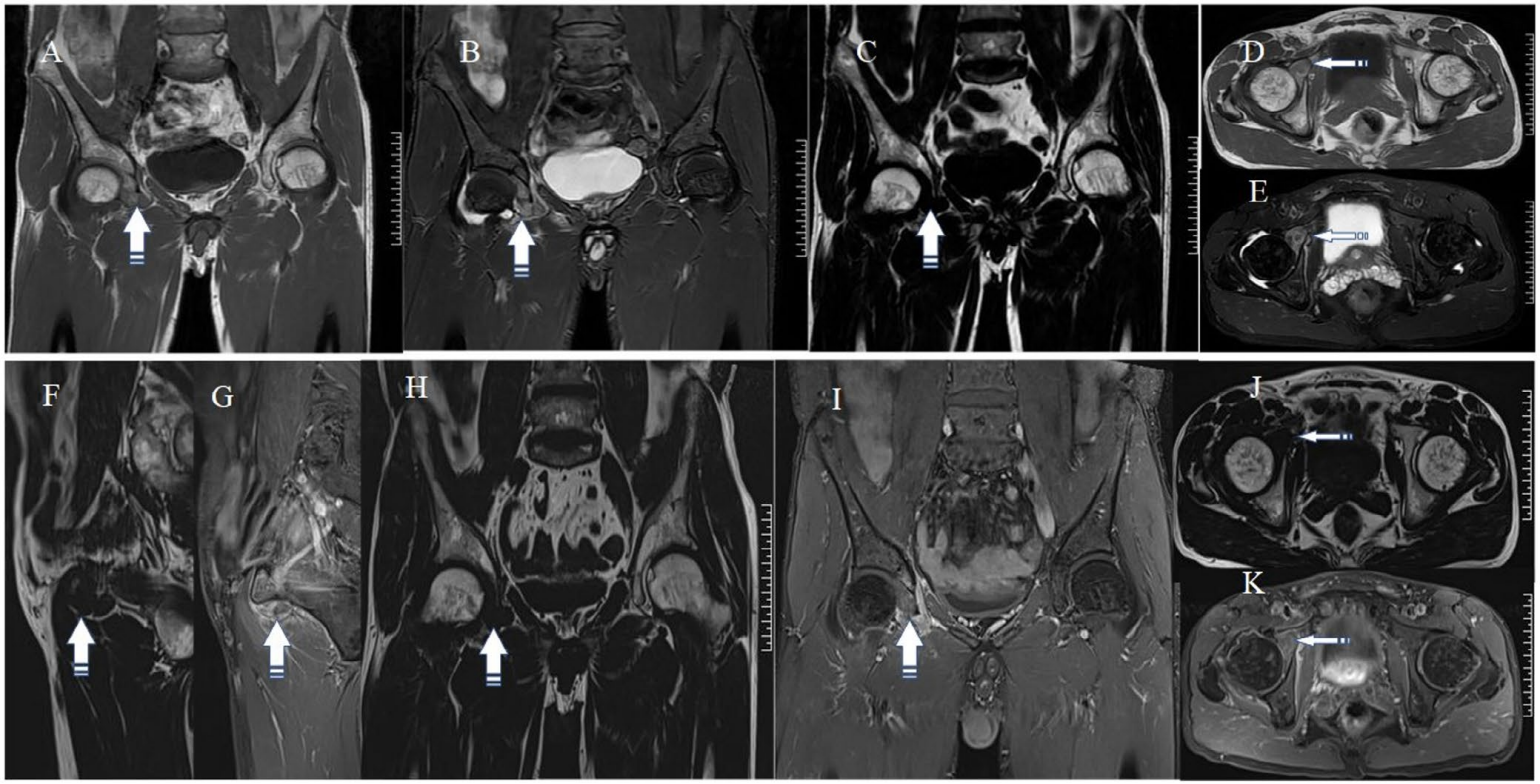
Magnetic resonance imaging of both hips. (A–E) Non‐contrast MRI, and (F–K) Contrast‐enhanced MRI. Mild bilateral hip joint effusion is present, more pronounced on the right, with synovial thickening. A patchy intra‐articular synovial lesion in the right hip demonstrates heterogeneous low‐to‐intermediate signal intensity on T1‐weighted images, heterogeneous signal intensity on T2‐weighted images, and heterogeneous enhancement after contrast administration. The lesion measures approximately 27 × 35 × 25 mm. Thinning of the adjacent anterior acetabular column and mild swelling of the right obturator externus muscle are also present. Arrows indicate the lesion.

**FIGURE 2:**
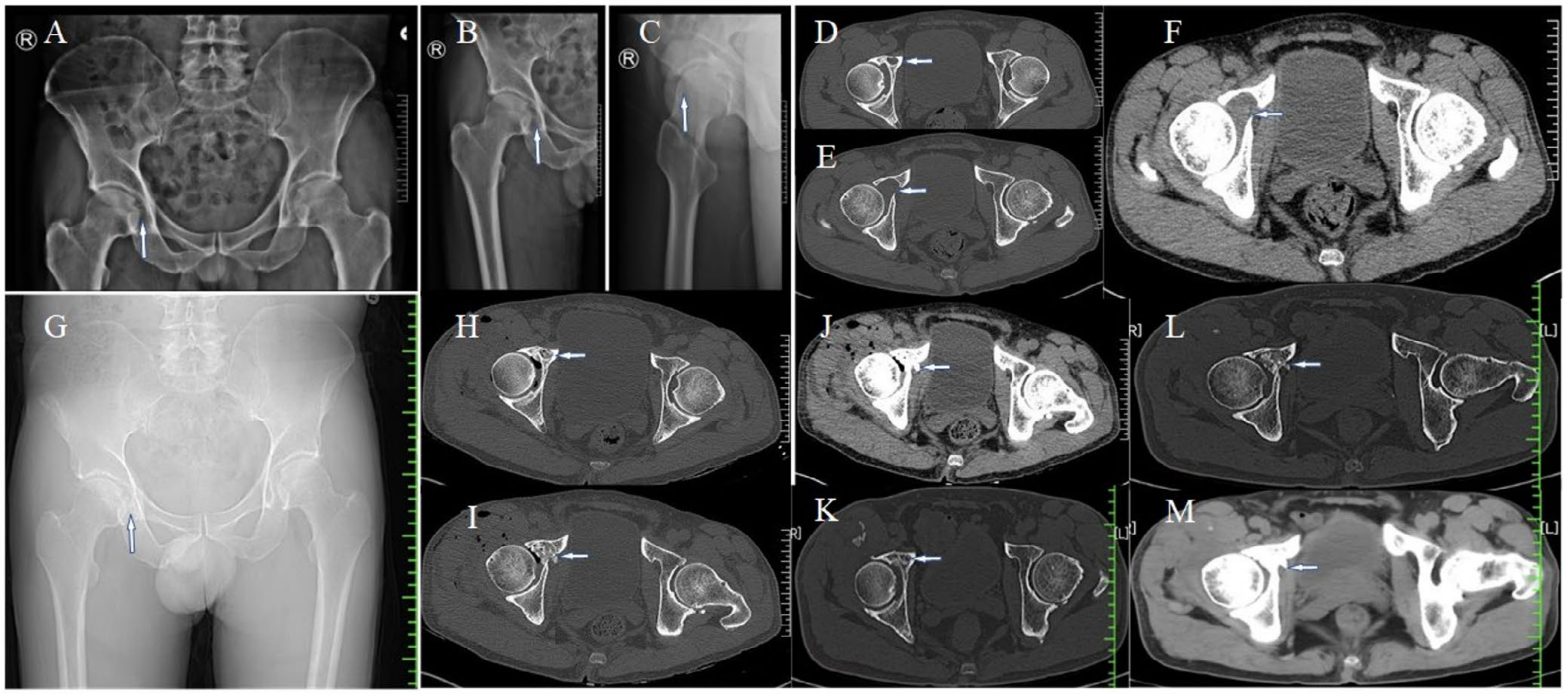
Conventional radiography and computed tomography (CT) of the right hip. (A–F) Preoperative images, (G–J) Immediate postoperative images, and (K–M) Images obtained at the 2‐month follow‐up. Preoperative CT demonstrates erosion and a partial osseous defect of the anterior and inferomedial acetabular wall. Immediate postoperative images demonstrate filling of the acetabular defect with an autologous iliac bone graft. Follow‐up images show maintained graft position and osseous incorporation. The preoperative plain radiograph shows no definite abnormality. Arrows indicate the acetabular lesion or reconstructed defect.

**FIGURE 3:**
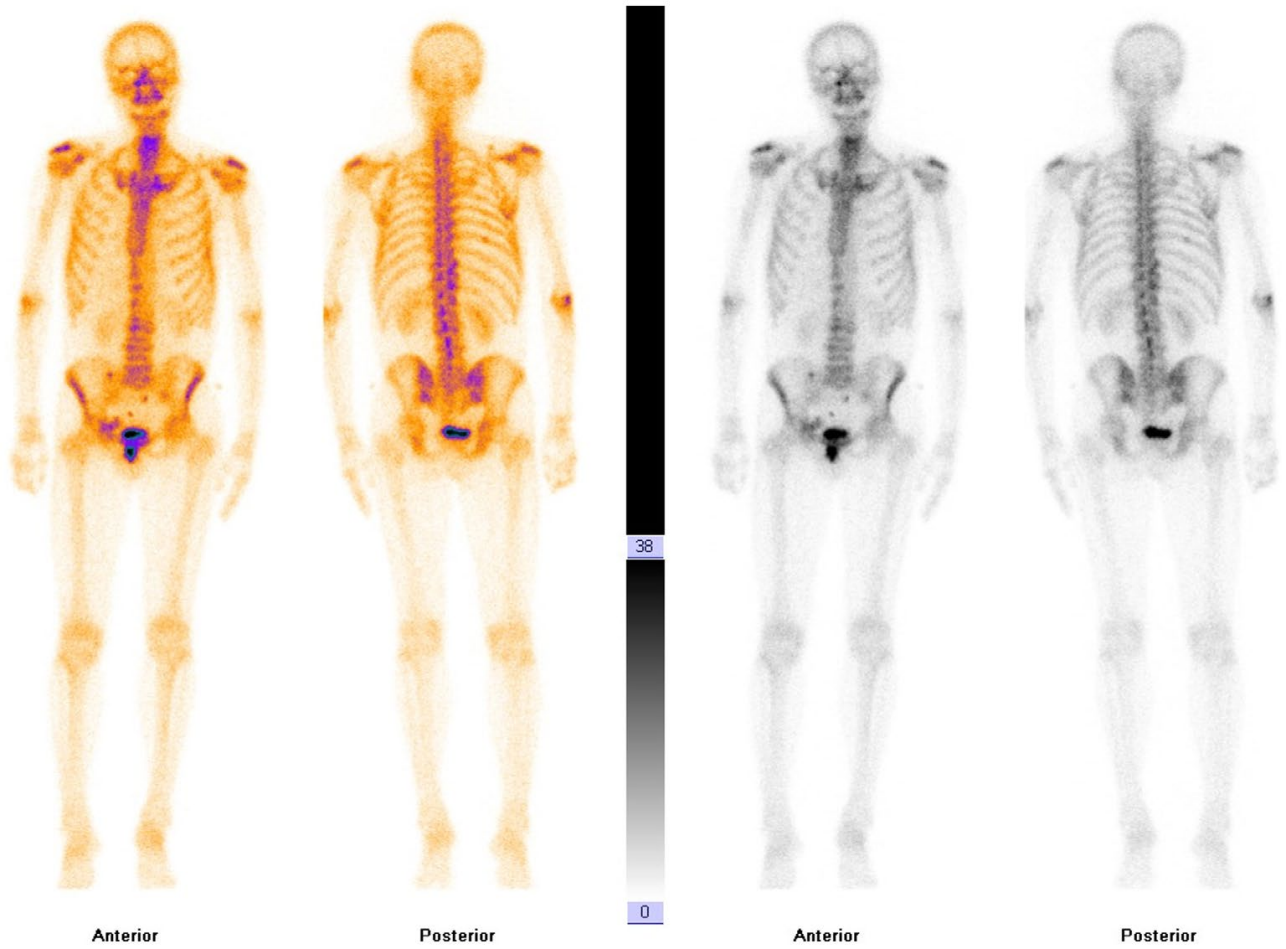
Whole‐body bone scan (anterior and posterior views) obtained 3 h after intravenous injection of 99mTc‐MDP. The skeletal structures are clearly visualized with good contrast. Heterogeneous radiotracer distribution is noted, with abnormal uptake observed in both shoulder joints, the upper thoracic spine, the right 5th–8th posterior ribs, both sacroiliac joints, and both hip joints. No other areas of abnormal radiotracer accumulation or photopenic defects are identified. Physiological excretion is seen in both kidneys and the urinary bladder. Focal areas of increased activity in the pelvic and perineal regions are attributed to urinary contamination.

Magnetic resonance imaging (MRI) of the hips demonstrated abnormal intra‐articular synovial tissue in the right hip, with mild bilateral joint effusion that was more pronounced on the right. The lesion showed heterogeneous low‐to‐intermediate signal intensity on T1‐weighted images, heterogeneous signal intensity on T2‐weighted images, and heterogeneous enhancement after contrast administration (Figure 1). The adjacent anterior acetabular column appeared thinned. Computed tomography (CT) performed after admission showed joint effusion, cystic low‐density intra‐articular lesions, and thinning and erosion of the anterior and inferomedial acetabular wall (Figure 2). Whole‐body bone scintigraphy demonstrated increased tracer uptake in both hips, sacroiliac joints, and shoulders, which was considered most likely to represent degenerative change (Figure 3). Laboratory testing showed a white blood cell count of 6.46 × 10^9^/L, a neutrophil percentage of 68.4%, a C‐reactive protein level of 34.20 mg/L, an erythrocyte sedimentation rate of 25 mm/h, and a procalcitonin level of 0.268 ng/mL; serum alkaline phosphatase and tumor marker levels were within the reference ranges. Given the recent febrile history, inflammatory abnormalities, and erosive acetabular lesion, the preoperative differential diagnosis included infectious or inflammatory synovitis, synovial chondromatosis, and other aggressive synovial or osseous lesions. Because the imaging and laboratory findings were not diagnostic, surgical exploration was undertaken after multidisciplinary discussion.

### 2.4 Therapeutic Intervention

**FIGURE 4:**
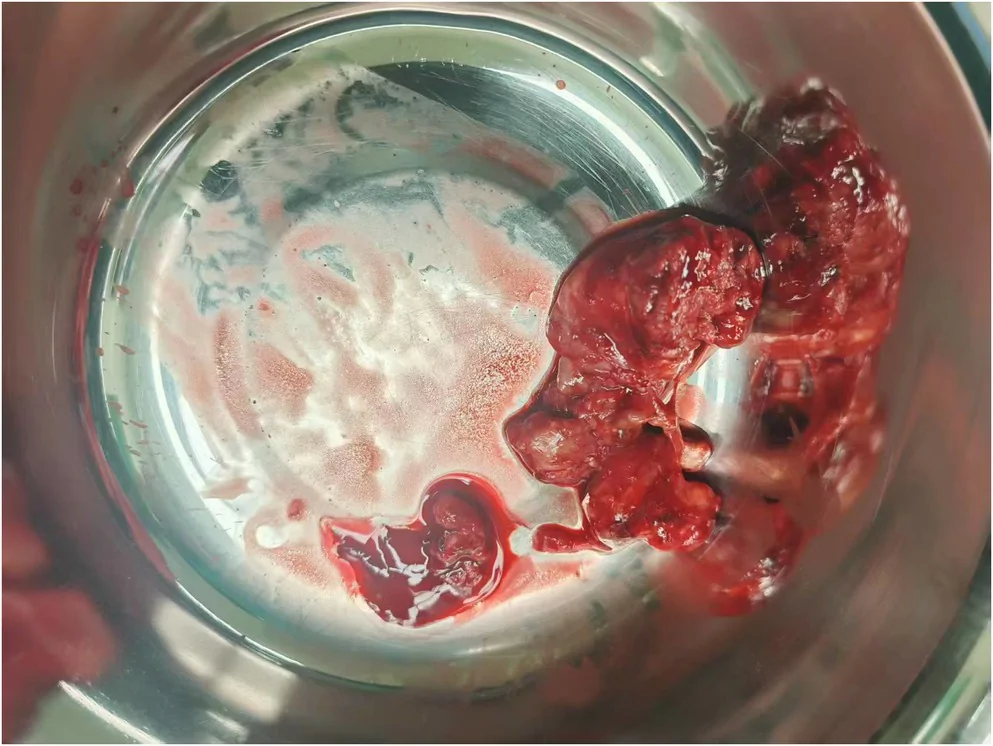
Intraoperative macroscopic findings. Multiple nodular soft‐tissue components measuring up to approximately 1.5 × 3.0 cm were identified.

Intraoperative inspection revealed abundant yellowish joint fluid and diffuse proliferation of synovium‐like tissue within the right hip joint. Irregular nodular lesions were present along the anteromedial and inferomedial acetabular wall. Complete excision of the identified intra‐articular lesions and involved synovial tissue was performed. The eroded inferomedial acetabular wall was curetted and treated with alcohol ablation, and the localized bone defect was reconstructed with an autologous iliac bone graft. Estimated intraoperative blood loss was approximately 150 mL, and the procedure was otherwise uneventful. Although the lesion contained multiple nodular components measuring up to approximately 1.5 × 3.0 cm, it was classified as diffuse‐type TGCT because of the widespread intra‐articular synovial involvement, acetabular erosion, and absence of a solitary encapsulated mass (Figure 4).

### 2.5 Histopathologic Diagnostic Confirmation

**FIGURE 5:**
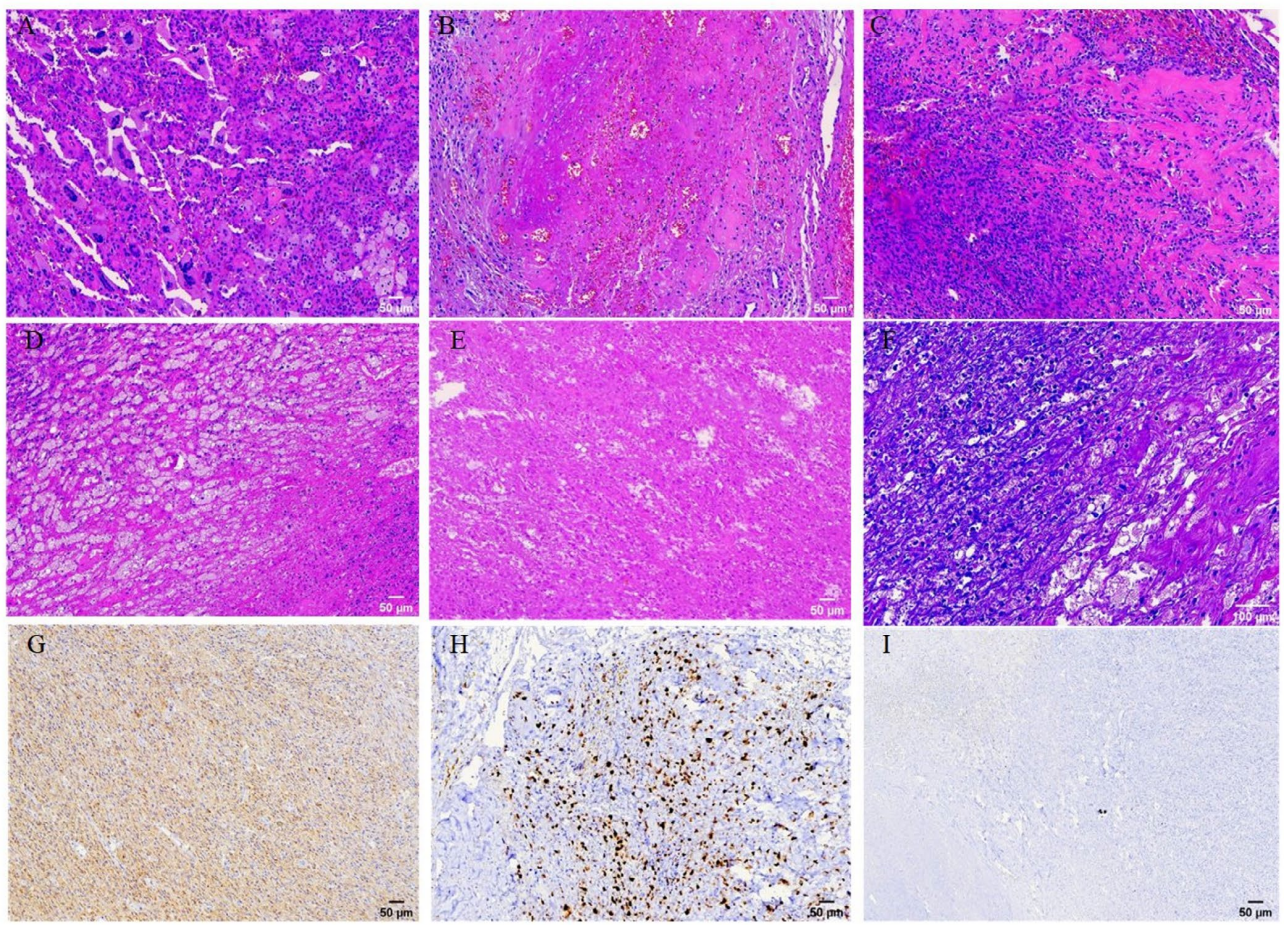
Histopathologic and immunohistochemical findings. (A) Nodular proliferation composed of mononuclear cells, osteoclast‐like multinucleated giant cells, and hemosiderin‐laden histiocytes (hematoxylin and eosin [H&E], ×20). (B) Focal coagulative necrosis with surrounding inflammatory cells and multinucleated giant cells (H&E, ×20). (C) Mononuclear cells arranged in fascicles and sheets with scattered osteoclast‐like multinucleated giant cells (H&E, ×20). (D) Aggregates of foamy histiocytes (H&E, ×20). (E) Coagulative necrosis surrounded by fibrous tissue and chronic inflammatory cells (H&E, ×20). (F) Intracytoplasmic hemosiderin deposition in histiocytes (H&E, ×40). (G) CD68 immunoreactivity in multinucleated giant cells and a subset of mononuclear cells. (H) Ki‐67 labeling index of approximately 5%–10%. (I) Focal S‐100 immunoreactivity in a subset of mononuclear cells.

Histopathologic examination showed proliferative synovium‐like tissue with focal cyst wall‐like structures lined by flattened to cuboidal cells. The stroma contained proliferative fibrous tissue, histiocytes, prominent congested vessels, diffuse chronic inflammatory cells, scattered neutrophils, and focal coagulative necrosis. Scattered and clustered foamy histiocytes and variable numbers of osteoclast‐like multinucleated giant cells were present. Hemosiderin granules were identified within the cytoplasm of mononuclear cells and histiocytes. Immunohistochemical staining showed strong CD68 positivity in multinucleated giant cells and a subset of mononuclear cells, focal S‐100 expression in a subset of mononuclear cells, and a Ki‐67 labeling index of approximately 5%–10%. Intraoperative joint‐fluid cultures were negative for bacterial and fungal growth. Integration of the diffuse intra‐articular growth pattern, characteristic histomorphology, imaging findings, and supportive immunohistochemical profile established the diagnosis of D‐TGCT (Figure 5).

### 2.6 Follow‐Up and Outcomes

The patient experienced marked pain relief immediately after surgery. On postoperative day 3, he developed fever (maximum temperature, 38.7°C), mild cough, and fatigue. Laboratory tests showed increased inflammatory markers, and chest CT demonstrated multiple patchy opacities in both lungs. In view of the respiratory symptoms and imaging findings, pulmonary infection was considered the most likely cause. The patient received intravenous cefuroxime and supportive treatment, after which his temperature normalized and systemic symptoms improved. At the 2‐week follow‐up, the incision was well healed, right hip pain had substantially improved, and the range of motion was better than before surgery. CT performed on the first postoperative day showed satisfactory filling of the acetabular defect by the bone graft, and CT at 2 months showed maintained graft position and osseous healing. No postoperative radiotherapy or systemic antitumor therapy was administered; the patient was managed with clinical and imaging surveillance. At the 6‐month follow‐up, he reported sustained pain improvement and had returned to independent daily activities and work, although mild limitation of right hip motion persisted.

### 2.7 Patient Perspective

The patient's perspective was not separately documented.

## 3 Discussion

D‐TGCT, previously termed pigmented villonodular synovitis, is a locally aggressive synovial neoplasm. Hip involvement remains diagnostically challenging because presenting symptoms are nonspecific, plain radiographs may be unrevealing, and cross‐sectional imaging findings can overlap with other conditions. In the present case, acute severe pain after a recent febrile illness, inflammatory abnormalities, and an erosive acetabular lesion complicated the preoperative assessment. Routine urine and stool tests, joint‐fluid culture, sputum culture, Haemophilus culture, sputum smear microscopy, and sputum DNA testing for *Mycobacterium tuberculosis* were negative. However, blood culture, formal synovial‐fluid analysis, Gram staining, and additional microbiologic testing were not performed. Prior cefuroxime exposure may also have reduced the sensitivity of subsequent cultures. Therefore, infection could not be excluded solely on the basis of negative microbiologic results. The final diagnosis was established by integrating the clinical course, imaging findings, intraoperative observations, and characteristic histopathologic features. Although osseous erosion is well recognized in hip D‐TGCT because the hip capsule has limited capacity to accommodate proliferative synovium, a destructive‐appearing acetabular lesion still raises concern for septic arthritis, inflammatory synovitis, synovial chondromatosis, or a more aggressive neoplastic process. The principal educational message of this case is that D‐TGCT should remain in the differential diagnosis of an unexplained erosive intra‐articular hip lesion.

MRI is the preferred imaging modality for mapping the extent of synovial disease. D‐TGCT typically shows low‐to‐intermediate signal intensity on T1‐weighted images and heterogeneous low, intermediate, or mixed signal intensity on T2‐weighted images because of variable proportions of fibrous tissue, lipid‐laden macrophages, fluid, and hemosiderin deposition [4, 6, 7]. MRI‐pathology correlation has also been emphasized in TGCT at other sites [8]. In our patient, MRI demonstrated diffuse intra‐articular synovial proliferation with heterogeneous contrast enhancement, whereas CT more clearly delineated the anterior and inferomedial acetabular erosion relevant to operative planning. Structured MRI assessment may also facilitate estimation of disease burden and follow‐up evaluation [7, 9]. Nevertheless, imaging alone could not reliably distinguish D‐TGCT from infectious, inflammatory, or other aggressive etiologies.

Histopathology remains the diagnostic gold standard. The characteristic microscopic pattern of D‐TGCT includes mononuclear cells, osteoclast‐like giant cells, foamy histiocytes, chronic inflammatory cells, and abundant hemosiderin deposition [4, 10]. Our case showed all of these features. Focal coagulative necrosis was interpreted as a secondary degenerative or ischemic change in the setting of extensive synovial proliferation and local inflammation. No marked cytologic atypia, atypical mitotic activity, sarcomatous overgrowth, or other features suggestive of malignant transformation were identified. Strong CD68 expression supported the histiocytic component of the lesion but was not diagnostically specific. Similarly, the relatively low Ki‐67 labeling index was compatible with low proliferative activity but did not independently establish the diagnosis. Focal S‐100 expression was regarded as nonspecific. At the molecular level, TGCT is associated with CSF1 overexpression and recruitment of macrophages through the so‐called landscape effect, a concept that also underpins CSF1/CSF1R‐targeted therapies [11, 12].

Surgical synovectomy and excision remain the mainstay of treatment when disease is technically resectable. However, diffuse hip involvement may be difficult to eradicate completely, and recurrence remains a recognized concern because of the complex anatomy of the joint. Adjuvant radiotherapy or systemic treatment directed against the CSF1/CSF1R axis may be considered in selected patients with residual, recurrent, or incompletely resectable disease [12, 13, 14]. In the present patient, gross excision and synovectomy were performed, and no postoperative radiotherapy was administered. Because the current follow‐up period remains short, this case should be interpreted primarily as an educational report on diagnostic evaluation and multidisciplinary management rather than as evidence of durable local control. Continued clinical and imaging surveillance is planned, and longer follow‐up is required to assess recurrence, joint function, and treatment durability.

## 4 Conclusion

In conclusion, D‐TGCT should be considered in patients with unexplained hip pain when MRI demonstrates proliferative synovial disease and CT shows erosive acetabular change, even when the clinical context raises concern for infection or inflammation. MRI and CT are complementary for defining soft‐tissue extent and osseous involvement, but definitive diagnosis depends on integration of characteristic histopathologic findings with the clinical and imaging features. Recognition of this diagnostic pitfall may help avoid misclassification of hip D‐TGCT as an infectious, inflammatory, or more aggressive destructive lesion.

## Author Contributions

**Wang Quanbing:** investigation, supervision, writing – review and editing, methodology, visualization. **Xing Huanhuan:** investigation, methodology, validation, software, formal analysis, writing – original draft, writing – review and editing. **Zhang Xing:** investigation, writing – original draft, visualization, software. **Yang Tao:** investigation, writing – original draft, data curation, formal analysis, software. **Zhang Jun:** investigation, writing – original draft. **Zhu Lei:** investigation, writing – original draft. **Li Kai:** investigation, writing – original draft. **Luo Lei:** investigation, writing – original draft, writing – review and editing. **Ao Feng:** investigation, writing – original draft. **Yang Wei:** investigation, funding acquisition, writing – review and editing, writing – original draft, supervision, resources, visualization, methodology.

## Funding

This work was supported by the Shiyan Renmin Hospital Hospital‐Level Guiding Project (syrmyn‐2026‐067), Talent Start‐up Funding Project of Hubei University of Medicine (2023QDJZR17), The Guiding Project of the Shiyan Municipal Science and Technology Bureau (25Y127), Natural Science Foundation of Hubei Province (2024AFB841).

## Ethics Statement

This study was a retrospective case report. Ethical approval was waived by the Institutional Review Board of Shiyan Renmin Hospital. Written informed consent was obtained from the patient for participation and for publication of this case report and accompanying clinical and imaging data.

## Consent

Written informed consent for publication of the patient's clinical details and images was obtained from the patient.

## Conflicts of Interest

The authors declare no conflicts of interest.

## Data Availability

The data that support the findings of this study are not publicly available due to ethical and privacy restrictions. The data are derived from a single patient case and contain potentially identifiable clinical, radiological, and pathological information. Further access to the data may be considered upon reasonable request to the corresponding author and with appropriate approval from the relevant ethics committee.
